# Influence of Y_2_O_3_ Doping on Phase Evolution and Dielectric Characteristics of ZrO_2_ Ceramics

**DOI:** 10.3390/mi15080938

**Published:** 2024-07-23

**Authors:** Lanfeng Gao, Yong Shao, Yangmei Xin, Dan Yang, Haizhong Zhang, Minmin Zhu, Li Zhang, Xiaoqiang Lu

**Affiliations:** 1 School of Advanced Manufacturing, Jinjiang Science and Education Park, Fuzhou University, Jinjiang 362200, China; 2 College of Physics and Information Engineering, Fuzhou University, Fuzhou 350116, China; 3 United Testing Services (Fujian) Co., Ltd., Shishi 362799, China

**Keywords:** Y_2_O_3_-doped zirconia ceramics, phase transformation, grain size, dielectric constant, breaking field

## Abstract

Improvements in phase stability and dielectric characteristics can broaden the applications of zirconia in ceramics. Herein, a series of Y_2_O_3_-stabilized zirconia (YSZ) ceramics are synthesized using solid-state sintering, followed by an investigation into their phase evolution, grain size, dielectric constant, and breaking field. As the Y_2_O_3_ content increases from 0 wt% to 4 wt%, the as-grown YSZ ceramics undergo a distinct phase transformation, transitioning from monoclinic to monoclinic + tetragonal and further to monoclinic + tetragonal + cubic, before finally returning to monoclinic + cubic. Significant changes occur in the internal microstructure and grain size of the ceramics as the phase composition alters, resulting in a reduction in grain size from 3.17 μm to 0.27 μm. Moreover, their dielectric constants exhibit an increasing trend as the Y_2_O_3_ content increases, rising from 3.92 to 13.2. Importantly, the dielectric breakdown field of these YSZ ceramics shows a similar variation to the phase evolution, ranging from 0.11 to 0.15 MV/cm. This study sheds light on the phase evolution and dielectric properties of YSZ ceramics, offering an efficient strategy for enhancing their dielectric performances.

## 1. Introduction

Zirconia (ZrO_2_) has garnered considerable attention owing to its diverse physicochemical properties, including low thermal conductivity, high fracture toughness, high ionic conductivity, and chemical and photochemical stability [1,2]. Its extensive research and application are widespread in diverse fields, such as oxygen sensors, thermal barrier coatings, corrosion-resistant materials, photonics, and as a potential alternative to SiO_2_ in high-k dielectric materials [3,4]. Zirconia exhibits three distinct spatial phase groups: monoclinic, tetragonal, and cubic phases [5]. However, due to the inherent phase instability and coexistence of multiple phases in ZrO_2_-based materials, their grain size and dielectric properties vary significantly, thus limiting their utility in the realm of dielectric and electronic devices. Hence, precise phase control is imperative for the production of high-quality zirconia ceramics.

Currently, several influential factors affect the phase transformation and stability of ZrO_2_, notably temperature, grain size, and additives. Typically, under normal temperatures and pressures, the monoclinic phase (M) remains thermodynamically stable below 1170 °C due to its lower symmetric structure. Upon reaching temperatures between 1170 °C and 2370 °C, the monoclinic phase undergoes a phase transformation, transitioning into the tetragonal phase (T) [6]. When temperatures exceed 2370 °C, further phase transformation occurs, ultimately leading to the cubic phase (C) [7]. Besides temperature, grain size also significantly impacts the phase transformation process of zirconia. When the grain size of pure zirconia is less than or equal to 30 nm, it automatically undergoes a phase change, acquiring metastable tetragonal zirconia. This is attributed to the existence of a nanoscale critical size for the transition from the tetragonal to the monoclinic phase. Furthermore, stable T and C phases can be obtained at room temperature by incorporating stabilizers, altering particle size, or introducing oxygen vacancy defects into the structure [8,9,10,11,12]. Oxide additives such as La_2_O_3_, CaO, and Y_2_O_3_ are commonly employed as stabilizers, with Y_2_O_3_ notably effective in inhibiting phase changes [5,13,14]. Most of the research conducted on YSZ ceramics has centered on elucidating its structural characteristics, mechanical properties, electrical conductivity, and dielectric constant [15,16,17,18,19]. Evidently, the transformation and coexistence of multiple phases result in corresponding variations in dielectric performance, consequently influencing their suitability for dielectric and electronic applications.

In this study, ZrO_2_ ceramics with varying Y_2_O_3_ content (ranging from 0 wt% to 4 wt%) were synthesized using solid-state sintering. Subsequently, the phase composition, grain size, dielectric constant, and breaking field of the YSZ ceramics were investigated. This study demonstrates that the concentration of yttrium oxide significantly influences the crystal phase and grain size of YSZ samples. Additionally, we observed notable enhancements in both the dielectric constants and the breaking field of these ceramics. This investigation offers valuable insights into the phase transformation and dielectric properties of YSZ ceramics, thus facilitating their application in dielectric and electronic contexts.

## 2. Materials and Methods

### 2.1. YSZ Ceramic Synthesis

Zirconia nanopowder (particle size: 20 nm to 70 nm; density: 5.89 g/cm^3^; purity: ≥99.99%, ZhongNuo Advanced Material (Beijing) Technology) and yttrium oxide nanopowder (particle size: 50 nm; density: 5.01 g/cm^3^; purity: ≥99.99%, ZhongNuo Advanced Material (Beijing) Technology) were utilized as the primary precursors in our solid-state sintering process. The precise proportions (0 wt%, 0.5 wt%, 1 wt%, 2 wt%, 3 wt%, and 4 wt% Y_2_O_3_ doping YSZ) of these powders were meticulously measured using a high-precision electronic scale and thoroughly mixed prior to the addition of the adhesive. The binder was formulated to constitute 7% of the powder mass. The main ingredients of the binder were pure water and polyvinyl alcohol. A ratio of pure water to polyvinyl alcohol of 19:1 was employed to achieve the required consistency and flow performance while minimizing any adverse effects on the final material properties. Subsequently, we combined the previously prepared mixture with the powder mixture in a mortar and stirred thoroughly on a heating platform set at 60 °C to ensure the uniform mixing of the two components. Upon completion of the mixing process, the mixture was transferred to a drying oven maintained at 70 °C for dehydration. Once the mixture was completely dry, it was ground evenly for 1–2 h to ensure the desired particle size was achieved.

In the pressure range of 5 MPa, the ground powder was carefully packed into the mold, and pressure was applied for a duration of 15 min to compact it into a cohesive block. Following the pressing process, the sample was transferred to a sintering furnace. Starting from ambient temperature, the temperature was gradually increased to 650 °C at a heating rate of 5 °C/min. We maintained this temperature for 2 h to complete the debinding process. Debinding serves to eliminate the binder and volatile components from the target material, establishing a foundational state for subsequent high-temperature sintering processes. Following debinding, we adjusted the heating rate and gradually increased the temperature from 650 °C to 1500 °C at a rate of 2 °C/min. This high-temperature sintering phase facilitated further fusion of the particles within the target material, resulting in the formation of a denser and more stable structure. The temperature was maintained at 1500 °C for 8 h to ensure complete final sintering. Finally, the temperature was gradually reduced to room temperature at a cooling rate of 10 °C/min. All ceramics underwent a polishing process. The initial smoothing process involved the use of 20 μm and 7 μm boron carbide abrasives. After grinding, 1 μm diamond powder was employed for coarse polishing, followed by fine polishing with 0.25 μm diamond powder. Finally, a protective paint was applied to chamfer the edges.

### 2.2. Material Characterization

The crystallization and microstructure of YSZ ceramics were thoroughly investigated using a range of comprehensive analytical techniques. X-ray diffractometry was conducted using a Shimadzu XRD-6000 instrument to analyze the crystalline structure of the YSZ ceramic samples, scanned at a speed of 5°/min over a 2θ range of 20° to 40°. Field emission scanning electron microscopy (FESEM) was employed utilizing a JEOL JSM-7600F microscope to examine the microstructure of the samples. All ceramic samples were tested under an accelerating voltage of 5 kV. Additionally, to study the chemical bonding properties, we utilized an ESCALAB Xi^+^ model X-ray photoelectron spectroscopy (XPS) system with an energy resolution of ≤0.5 eV.

Furthermore, a composite electrode consisting of 15 nm Ni and 45 nm Au was prepared on the ceramic surface using Angstrom Engineering’s electron beam evaporation equipment. The dielectric properties of the resulting YSZ samples were evaluated using an impedance parameter analyzer (Agilent 4294A) equipped with a two-point probe over a frequency range of 0 Hz–1 MHz. This comprehensive suite of analytical techniques enabled a thorough examination of both the structural and chemical properties as well as the dielectric characteristics of the YSZ ceramics, such as dielectric constant and breakdown field.

## 3. Results and Discussion

**Figure 1:**
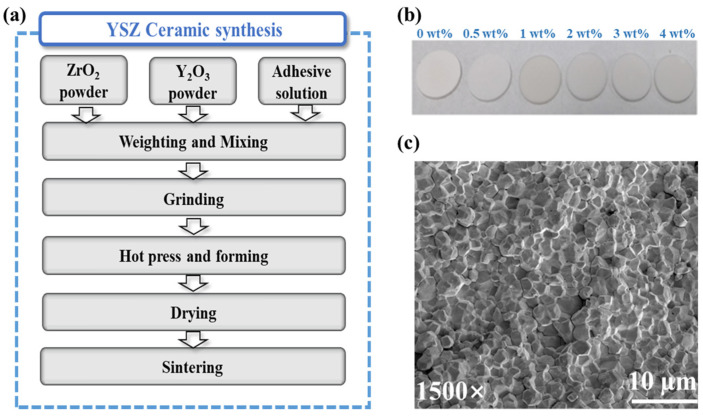
(**a**) The YSZ ceramic preparation process. (**b**) Photographs of the as-prepared YSZ ceramic. (**c**) SEM image of ZrO_2_ ceramics.

Zirconia-based ceramics were synthesized using the solid-state sintering technology [20], owing to cost-effectiveness, precise component control, and the capacity of mass production, as illustrated in the flow chart in Figure 1a. Through this fabrication process, we successfully achieved the production of relatively dense YSZ ceramics. The densities of YSZ ceramics doped with Y_2_O_3_ from 0 wt% to 4 wt% were 93.1%, 93.8%, 93.3%, 93.7%, 93.2%, and 94.1% of the theoretical density of fully dense YSZ, respectively.

Following double-size chemical and mechanical polishing, photographic images of YSZ samples with various proportions of doped Y_2_O_3_ (0–4 wt%) were obtained (Figure 1b), allowing for subsequent dielectric characterizations. Notably, the images revealed smooth and flat ceramic surfaces. Furthermore, a microscopic examination of the sample surface was conducted to assess its morphology. Figure 1c illustrates the surface morphology of the YSZ ceramic with 0 wt% Y_2_O_3_ content. Despite the presence of some pores, the particles on the ceramic surface demonstrate a predominantly compact arrangement. This dense interconnection between grains enables ceramics to effectively withstand external forces and uniformly distribute stress, thereby minimizing the damage caused by fracture or deformation. Consequently, this compact arrangement significantly enhances the overall stability of the ceramic material [21].

**Figure 2:**
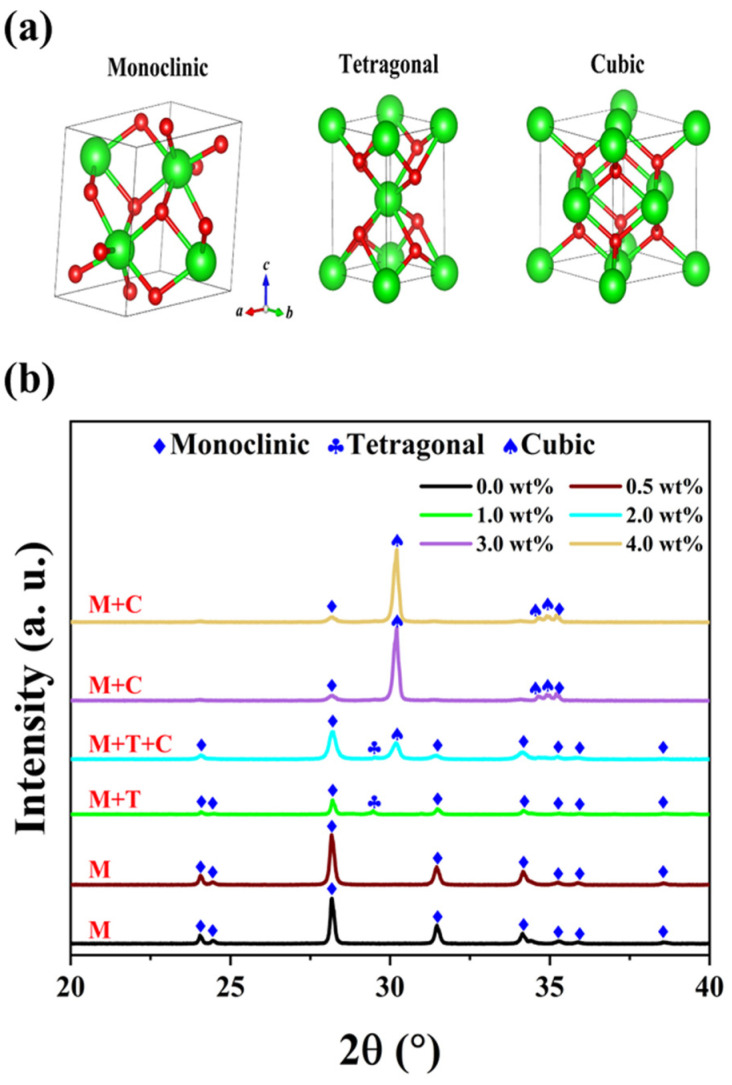
(**a**) Crystal structures of ZrO_2_. (**b**) XRD patterns of YSZ samples with different contents of Y_2_O_3_ sintered at 1500 °C.

The physical properties and structural characteristics of oxide ceramics strongly depend on their phase composition [22]. Generally, zirconia commonly exhibits three space groups: monoclinic (M), tetragonal (T), and cubic (C), as illustrated in Figure 2a. The monoclinic phase exists stably under normal conditions, while the tetragonal and cubic phases occur under specific conditions, enhancing the stability of ZrO_2_ ceramics.

Figure 2b demonstrates a distinct transformation between the three phases. The undoped YSZ ceramic (ZrO_2_) possesses only a monoclinic phase, retaining this structure even when the Y_2_O_3_ content is below 1 wt%. However, as the content of doping Y_2_O_3_ continues to increase beyond 1 wt%, a characteristic peak of the T phase emerges near 29.4°. The atomic radius of yttrium is approximately 180 pm, which is slightly larger than that of zirconium (160 pm), facilitating more efficient filling of vacancies in the crystal lattice by yttrium atoms and thereby stabilizing the formation of the T phase [23,24]. With further increases in yttrium oxide content, the larger size of yttrium atoms plays a pivotal role in optimizing the crystal structure. Consequently, when the doping content reaches 2 wt%, the coexistence of M, T, and C phases is observed. Interestingly, when the Y_2_O_3_ content exceeds 3 wt%, the characteristic peak associated with the T phase disappears. The doping of Y_2_O_3_ induces significant distortion in the coordination layer, leading to the formation of a stable solid solution. The lattice structure of this solid solution comprises the mixed monoclinic and cubic phases, enabling the zirconia material to maintain a metastable state at room temperature and prevent eutectoid decomposition during rapid cooling processes [25,26]. The underlying mechanism for the series of phase transitions in zirconia with yttria addition is the partial substitution of Zr^4+^ ions in its lattice by Y^3+^ ions. As the Y_2_O_3_ doping concentration increases, the YSZ ceramic evolves from the M phase to the M + C phase, accompanied by the formation of oxygen vacancies [27].

**Figure 3:**
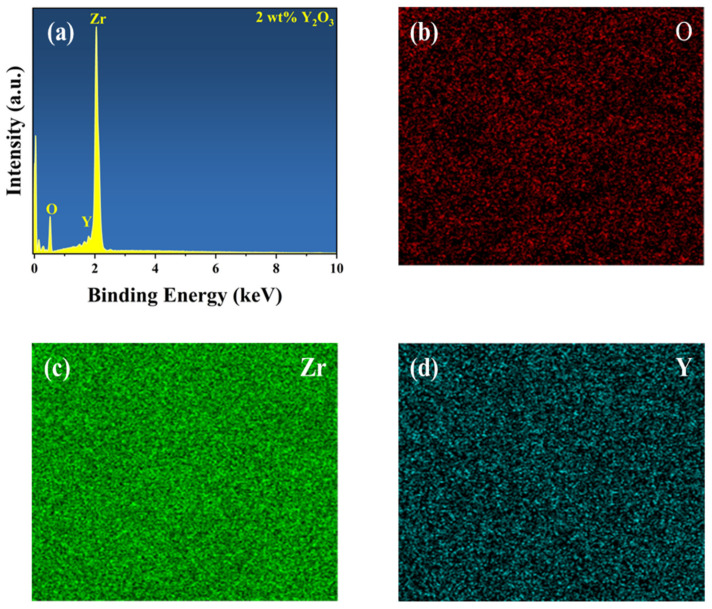
(**a**) EDS spectrum of YSZ ceramics with 2 wt% Y_2_O_3_ content. Elemental mapping of (**b**) O, (**c**) Zr, and (**d**) Y.

**Figure 4:**
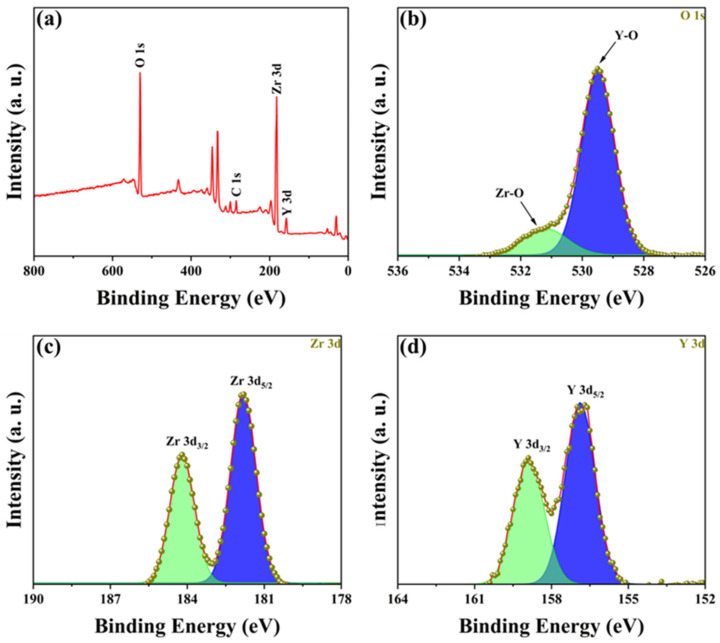
(**a**) XPS spectrum of YSZ ceramics with 4 wt% Y_2_O_3_ content. HRXPS spectra of (**b**) O 1s, (**c**) Zr 3d, and (**d**) Y 3d.

To determine the composition of the YSZ ceramic and reveal its surface element composition and electronic structure information, further analysis was performed using EDS and XPS. Figure 3 presents the EDS spectrum and element distribution profile of YSZ ceramics with a 2 wt% Y_2_O_3_ content. An inspection of the figure reveals an even dispersion of Zr, Y, and O elements, indicating the uniform composition of the YSZ ceramics. Figure 4a presents the full spectrum scan results of YSZ, revealing the presence of elements such as Zr, Y, and O on the sample surface. Content analysis indicates that the content of Y_2_O_3_ is approximately 4 wt%. Figure 4b depicts the high-resolution XPS (HRXPS) spectra of O 1s. Two remarkable peaks located at 529.5 eV and 530.5 eV are present, corresponding to the lattice oxygen of both Zr-O and Y-O [28,29]. HRXPS spectra of both Zr 3d and Y 3d exhibit a pair of split peaks, as shown in Figure 4c,d. The Zr 3d peaks at 181.8 eV and 184.2 eV were assigned to 3d_5/2_ and 3d_3/2_, while the Y 3d peaks at 156.9 eV and 159.0 eV were ascribed to 3d_5/2_ and 3d_3/2,_ which is in agreement with the reported literature [30]. Since the Y_2_O_3_ content is too low, the reason for using XPS is to further analyze and determine whether the phase change in the ceramic is caused by Y_2_O_3_ doping and not the sintering process.

**Figure 5:**
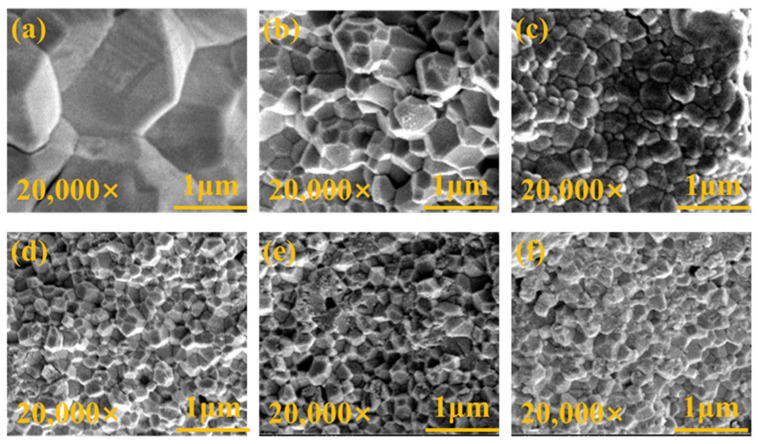
Surface morphologies of YSZ ceramics with (**a**) 0 wt%, (**b**) 0.5 wt%, (**c**) 1 wt%, (**d**) 2 wt%, (**e**) 3 wt%, and (**f**) 4 wt% Y_2_O_3_ contents.

**Figure 6:**
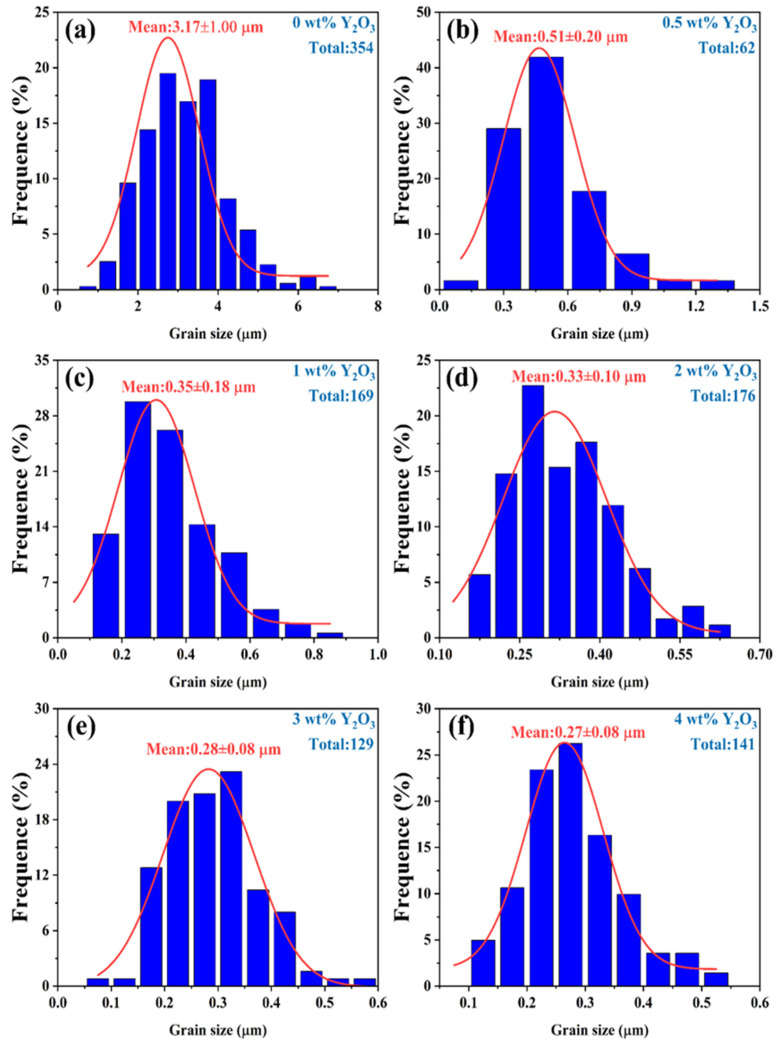
The grain size of YSZ ceramics with different Y_2_O_3_ doping contents at a sintering temperature of 1500 °C.

In the investigation of the correlation between YSZ phase transformation and grain size, we employed SEM to conduct meticulous observations of the surface morphology of the samples. Figure 5 depicts high-quality SEM images of YSZ ceramics with varying Y_2_O_3_ content ranging from 0 wt% to 4 wt%. All ceramic samples exhibit densified surfaces and consist of numerous grains of varying sizes. Statistical analysis of the grain sizes in these ceramics is presented in Figure 6.

Due to the irregularity of the grains, the diagonal method was used to mark them when using ImageJ software for statistics. Notably, the YSZ sample without Y_2_O_3_ doping demonstrates a large grain size of 3.17 μm. As the Y_2_O_3_ content increases from 0.5 wt% to 4 wt%, the average grain size of the YSZ ceramic targets exhibits an overall downward trend, decreasing from 0.51 μm to 0.27 μm. This decrease in average grain size suggests that the grain growth of YSZ ceramics is somewhat inhibited during the process of increasing the Y content. This inhibition may be attributed to the distribution, chemical state, and interaction of Y with other elements in YSZ [31]. Table 1 provides a comprehensive overview of the phase structure, grain size, and dielectric properties of YSZ ceramics.

**Figure 7:**
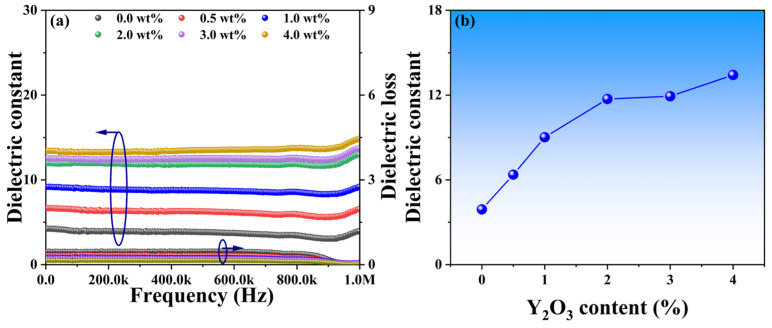
(**a**) Dielectric constants and losses of YSZ ceramics with varying Y_2_O_3_ contents as a function of frequency. (**b**) Dielectric constant vs. Y_2_O_3_ doping content at the frequency of 10 kHz.

Phase distributions and microstructure characteristics may play a crucial role in the dielectric performance of these YSZ ceramics. Figure 7a illustrates the dielectric constant of YSZ samples with varying Y_2_O_3_ content across a broad frequency range from 0 to 1 MHz. All dielectric constants exhibit a slight decrease as frequencies increase, followed by a significant increase at around 900 kHz. The gradual decrease in dielectric constant with increasing frequency is attributed to the reduction in space charge polarization effects [32]. Moreover, the observed increase in dielectric constant at 900 kHz may stem from either a relaxation process within the frequency range or a decrease in friction against dipole motion with increasing frequency [33]. Specifically, as the Y_2_O_3_ content increases from 0 wt% to 4 wt%, the dielectric constants of these ceramics at a frequency of 10 kHz vary from 3.9 to 13.4, suggesting that the addition of Y_2_O_3_ has the potential to enhance dielectric properties (Figure 7b). Surface morphology with smaller grains, as evidenced by Figure 5, facilitates a more uniform distribution of the internal electric field, resulting in a more stable electric field distribution. A stable electric field is crucial for achieving optimal dielectric properties, as it can minimize energy loss within the electric field and thereby improve the dielectric constant.

**Figure 8:**
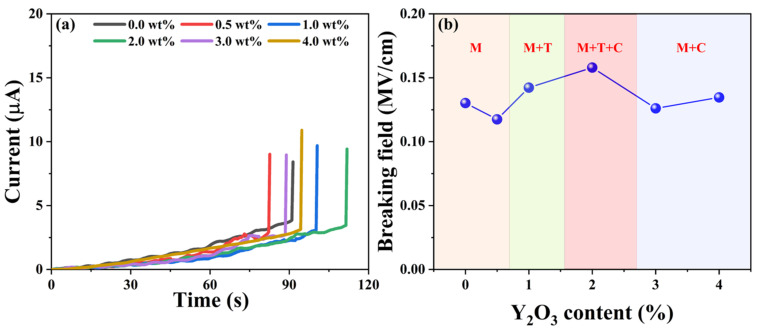
(**a**) Current vs. time curves of YSZ ceramics with varying Y_2_O_3_ content. (**b**) Breaking field vs. the doping levels of Y_2_O_3_.

Dielectric breakdown occurs when the electrical potential across a dielectric material surpasses its dielectric strength, leading to partial ionization [32]. Figure 8a illustrates the dielectric breakdown fields of the as-synthesized YSZ ceramics, showcasing a parallel trend with the phase evolution. As depicted in Figure 8b, initially, the breakdown field remains at a low level below 1 wt% of the doping Y_2_O_3_ content, corresponding to the pure M phase. However, as the doping content varied from 1 wt% to 2 wt%, the dielectric breakdown field significantly increased, reaching a maximum value of 0.15 MV/cm. The low breakdown electric field observed in the material could be attributed to several factors. Firstly, the thickness of the sample, which ranges from 1.4 mm to 1.7 mm, increases the likelihood of defects occurring. Furthermore, the presence of pores in the ceramic material distorts the local electric field, favoring the initiation of a breakdown path. Both of these factors contribute significantly to the observed decrease in breakdown field strength [34,35]. Notably, at a Y_2_O_3_ content of 2 wt%, the ceramic achieves a remarkable three-phase coexistence state, validated by XRD analysis in Figure 2. With a further increase in the doping percentage of Y_2_O_3_, the breakdown field of YSZ gradually diminishes due to the phase transformation from three-phase coexistence (M + T + C) to two mixed phases (M + C). Hence, the presence of multiple phases coexisting in ceramics proves particularly beneficial for enhancing the breakdown of the electric field, suggesting an efficient strategy for optimizing the electrical properties of YSZ ceramics.

**Figure 9:**
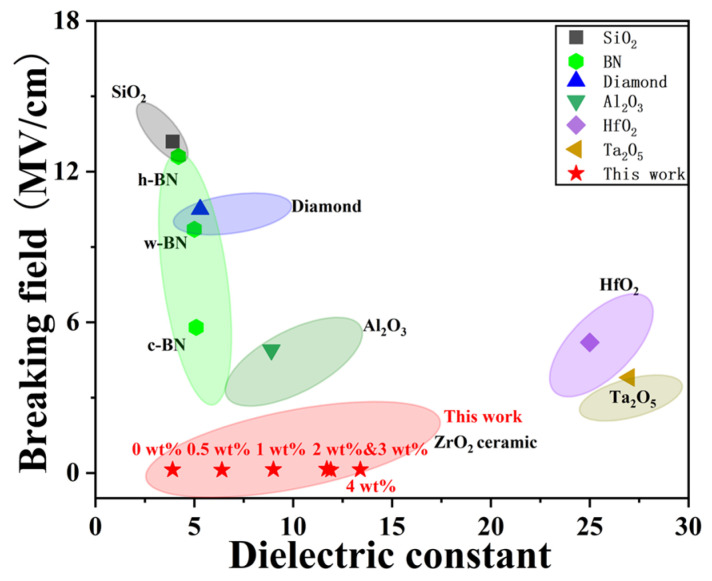
The breakdown field and dielectric constant of YSZ ceramics in comparison with common dielectric materials.

Common dielectric layers such as SiO_2_, Al_2_O_3_, HfO_2_, and Ta_2_O_5_ have found widespread application in electronic devices owing to their excellent dielectric constants, high breakdown electric fields, and wide band gaps [36]. Figure 9 summarizes the breakdown field and dielectric constant of our YSZ ceramics in comparison with various dielectric materials. Although Al_2_O_3_, HfO_2_, and Ta_2_O_5_ exhibit large dielectric constants (>10), they demonstrate a smaller breakdown field compared to other dielectric counterparts. Interestingly, hexagonal-BN (h-BN), wurtzite-BN (w-BN), and cubic BN (c-BN) consistently maintain a low dielectric constant of 3–5 while sustaining a dielectric breakdown field within the range of 5.8 to 12.6 MV/cm [32,36,37]. Similarly, SiO_2_, commonly used as the mainstream dielectric layer in CMOS, reveals a high breakdown field of 13.2 MV/cm and a dielectric constant of 3.9. Importantly, our YSZ ceramics exhibit a distinct dielectric breakdown field of 0.1–0.2 MV/cm while maintaining a broad dielectric constant ranging from 3.9 to 13.4. By employing these bulk ceramics as targets for pulsed laser deposition (PLD) and sputtering, the resulting thin films can achieve a high breakdown field of ~4 MV/cm or above [38]. This indicates promising potential for replacing both conventional dielectric materials, such as SiO_2_ and Al_2_O_3_, as well as emerging dielectric materials, including BN.

## 4. Conclusions

In summary, YSZ ceramics with varying Y_2_O_3_ content ranging from 0 wt% to 4 wt% were fabricated using solid-state sintering. Their phase evolution, grain size, dielectric constant, and breakdown field were systematically investigated. This work analysis reveals that as the Y_2_O_3_ content increases from 0 wt% to 4 wt%, the as-synthesized YSZ ceramics undergo a series of distinct phase transformations. The grain size of these ceramics significantly decreases from 3.17 μm to 0.27 μm with alterations in Y_2_O_3_ content, while their dielectric constants demonstrate an increasing trend, ascending from 3.92 to 13.2. Furthermore, the dielectric breakdown field of these YSZ ceramics exhibits a similar variation to the phase evolution, ranging from 0.11 to 0.15 MV/cm. This study not only offers insights into efficient strategies for enhancing ceramics’ dielectric performance but also highlights the considerable potential of YSZ in electronic power devices.

## Figures and Tables

**Table 1:** Phase structure, grain size, and dielectric properties of YSZ ceramics.

Materials	Phase	Grain Size (μm)	*ε* at 10 kHz	V_b_ (MV/cm)
0.0 wt%Y_2_O_3_-YSZ	M	3.17 ± 1.00	3.9	0.13
0.5 wt%Y_2_O_3_-YSZ	M	0.51 ± 0.20	6.4	0.11
1.0 wt%Y_2_O_3_-YSZ	M + T	0.35 ± 0.18	9.0	0.14
2.0 wt%Y_2_O_3_-YSZ	M + T + C	0.33 ± 0.10	11.7	0.15
3.0 wt%Y_2_O_3_-YSZ	M + C	0.28 ± 0.08	11.9	0.12
4.0 wt%Y_2_O_3_-YSZ	M + C	0.27 ± 0.08	13.4	0.13

## Data Availability

Data will be made available on request.
